# Why some children from poor families do well—an in-depth analysis of positive deviance cases in Singapore

**DOI:** 10.1080/17482631.2018.1563431

**Published:** 2019-01-29

**Authors:** Chelsea J.Y. Cheang, Esther C. L. Goh

**Affiliations:** a Early Intervention Centre, Asian Women’s Welfare Association; b Department of Social Work, National University of Singapore, Singapore

**Keywords:** Children, agency, audio-diary methods, low-income families, positive deviance

## Abstract

**Purpose**: Research documents that children from low-income families face higher risks in many areas of their development including academic performance. However, some children from low-income homes excel academically despite their disadvantaged environment. **Method**: Using Positive Deviance methodology (PD), audio-diary and interview data were collected from ten children who scored at least 70 percentile in school examinations in spite of their financial deprivation.**Results**: This paper uncovers specific dimensions of agency in these children that stemmed from the relational contexts they had with their mothers. Combining the PD methodology and sensitizing lens from Social Relational Theory, this study provided evidence that PD children are connected agents within their family. It suggests that children's awareness of their family circumstances motivated them to work hard and enabled them to devise creative ways to manage their limited financial resources.**Conclusions**: The findings challenge dominant discourses on poor children as passive victims and suggest new ways for practitioners to examine the relationship contexts that support children's capacity as agents rather than focusing on individual traits.

## Introduction

The impact of poverty on the lives of children and their families is devastating, long lasting, and generational (Carter, 2014). About 20% of the world’s children live in extreme poverty (UNICEF, 2017). Children are often viewed as the greatest victims because they enter poverty by virtue of their family’s financial situation which they are powerless to alter (Brooks-Gunn & Duncan, 1997). Poverty adversely affects children’s life chances and well-being in terms of physical, emotional, social, and cognitive abilities and school achievement (Brooks-Gunn & Duncan, 1997; Dahl & Lochner, 2012; Gruber, 2009; Lee, 2011). Families with a lower socioeconomic status (SES) have been found to have fewer financial and educational resources and less access to social and cultural capital. This deficit in family resources is adversely associated with children’s well-being (Bradley & Corwyn, 2002; Claro, Paunesku, & Dweck, 2016; Watkins & Howard, 2015).

In Asia, particularly Singapore, education is the key route for social mobility (Ong & Cheung, 2016). As a result, children from low-income families who perform poorly in school have to work harder to catch up with their peers or risk remaining within the poverty cycle. The Singapore government has in recent years implemented policies which aim to help children from poor families be on a level playing field with their more well-to-do peers. Financial assistance schemes are available to these families to help with their school expenses, in the hope that the children do not lag behind their peers (Ng, 2014). In spite of these efforts to lower external barriers, it is not uncommon to find children from low-income families still struggling to perform in school, and therefore high rates of failure remain (Ng & Li, 2014). Although some scholars (Teo, 2018) assert that social policies have thus far been inadequate in supporting low-income families, we acknowledge the need for continual policy efforts to enhance environmental protective factors for children from low-income families to do better academically. In this paper, we propose that social workers cast their attention simultaneously on macro level policy changes as well as on micro level opportunities that exist within low-income families. We underscore the potential value of solutions provided by children who grow up in resource-deprived home environments and yet outperform their peers academically.

A small body of research has focused on academic resilience among economically disadvantaged students. These studies have identified external factors including social support in home, school, and the community (Martin, 2002; Wang, Haertel, & Walberg, 1997, Williams & Bryan, 2013; Wyner, Bridgeland, & DiIulio, 2007) that contributed to academic success. In particular, the involvement of parents has been found to buffer against the effects of poverty (Barajas, Philipsen, & Brooks-Gunn, 2008; Jowkar, Kojuri, Kohoulat, & Hayat, 2014; Williams & Bryan, 2013). These studies tended to take the perspectives of parents, teachers, and other adults. The perspectives of children are rarely considered in educational research (Bishop & Pflaum, 2005). Since children are the primary stakeholders of their own learning process (Lincoln, 1995), and they may have unique viewpoints and values about their own education, it is critical to examine children’s perspectives (Downey, 2014; Mazzoni & Harcourt, 2013).

The purpose of the present study is to explore children’s perspectives on their own academic success despite facing poverty in Singapore. Academic performance provides us with a window for us to examine how children act in the capacity of human agents in the context of adverse family circumstances. We assumed that since education is much valued by the community, children’s achievement or the lack of it will alert us to their sense of mastery over their situations. By listening to the children and examining their perspectives, thought processes, motivations, and actions in navigating their daily challenges and interactions with their environment, we hoped to draw out children’s own views of the individual capabilities and relational resources that support their efforts as agents of their own academic performance. We were interested in how academically successful children perceived their own efforts and resources for managing the stressors, deficiencies, and challenges stemming from financial constraints in their home. To achieve this, we used two conceptual tools, positive deviance (PD) methodology, which provides a framework for identifying participants who succeed despite adversity, and social relational theory (SRT), which provides a framework for exploring these participants’ actions as agents in their own academic achievement.

### PD methodology for researching human agency

PD is a bottom-up methodology which identifies and learns from those who demonstrate successful performance on an outcome of interest. It assumes that problems can be overcome using solutions that already exist within the community (Baxter, Taylor, Kellar, & Lawton, 2015; Bradley et al., 2009; Marsh, Schroeder, Dearden, Sternin, & Sternin, 2004). Although typically employed in the context of improving nutrition (Marsh et al., 2004) or tackling behavioural issues relating to health care (Baxter et al., 2015), PD methodology is also applicable when a problem is widespread and the minority in the community exhibits the desired behaviours (Fowles, Hendricks, & Walker, 2005; Sternin, 2002). In this paper, we extended this methodology to explore whether another solution within the community lies in the agency of children themselves. We used PD methodology to identify a rare sample of children from low-income families who exhibited academic success against prevalent odds of poor academic performance stemming from poverty (Cunha, Heckman, Lochner, & Masterov, 2006; McLoyd, 1998), and attained academic performance beyond the 70th percentile among their peers.

We argue that PD methodology is a useful strategy for identifying children’s successful manifestation of agency to meet academic goals despite adversity. First, it provides a conceptual approach in selecting a theoretically interesting sample of children who demonstrated impressive academic results despite poverty. Second, the close examination of these children’s motivations, thoughts and actions in the process of interacting with their challenging environments may help identify those features of children’s strategic actions as agents that are implicated in positive outcomes

We used SRT (Kuczynski & De Mol, 2015) as a conceptual framework to guide us in the collection and analysis of data in order to understand what children actually did that contributed to their success.

### Sensitizing lens provided by SRT

SRT provides a dialectical framework and dynamic sensitizing lens in understanding human agency by accessing the *individual agentic qualities* of children within the *relationship contexts* of their families. It provides a nuanced yet contextualized analysis of how PD children exercise agency to exploit intra-familial and external environments.

SRT proposes three components to consider the complexity of interdependent motivations to maintain *autonomy, construction*, and *actions* that feed into causal processes (Kuczynski, 2003). *Autonomy*, the motivational aspect of agency, refers to a person’s need to feel effective in their interaction with others and to protect their actions when blocked by others. *Construction* refers to a person’s capacity to construct meanings for their own behaviour and that of others. *Action* refers to a person’s capacity to have an effect on other people and the environment by acting or refraining from acting. Such partition of the construct of agency is purely for analytical purpose because, in practice, these components are experienced in an interdependent embodied way (De Mol, Reijmers, Verhofstadt, & Kuczynski, 2018; Kuczynski & De Mol, 2015).

In addition, people’s sense of agency is inherently relational, because how they act and perceive themselves as agents depends on the social relationship contexts in which they exercise their agency (De Mol et al., 2018). The constructs of *isolated* versus *connected agents* offered by SRT helps examine the nature of relationship contexts in which the PD children are embedded. When children feel connected with their parents they may have a higher sense of being effective as agents because they feel that they matter to their parents, that parents will be receptive to their influence, and can expect their actions to be supported by parents. When children are disconnected in their relationships, they may feel that they do not matter to their parents or that their parents will not be responsive to or supportive of their actions as agents. Children in such disconnected relationship contexts are agents but tend to exercise their agency as *isolated agents* by acting in extreme, unconstructive, and self-serving ways. Relationally connected children, on the other hand, have a history of involved, responsive and intimate relationships and have a relatively high stake in their relationships with parents and others in their social networks. As *connected agents* they are more likely to have their expression of agency both supported by others and constrained by a desire to protect their valued relationships (Kuczynski & De Mol, 2015).

In summary, the goal of this paper is to explore the experiences of a carefully selected sample of children identified by PD methodology to unpack the agency demonstrated by them in how they overcame financial deprivation and devised solutions that contributed to their own success.

## Method

The study sets out to identify children within middle to late childhood, specifically between 9 and 13 years old, who exhibited the characteristics of PD. PD was operationalized as children who attained an overall academic performance at or beyond the 70th percentile in the most recent school examinations and who also came from low-income families. A household was classified as *low-income* when it had a per capita gross income not exceeding US$465 and, hence, was eligible to receive support from the School Pocket Money Fund (SPMF) or the Ministry of Education Financial Assistance Scheme (MOE FAS). Because very few children met these two criteria for PD, we cast our recruitment nets more widely by approaching social workers from three community-based Family Service Centres (FSCs) serving the needy populations in different locations, and requesting that they help to identify cases they served that fit into the sampling criteria of this study. The social workers from the FSCs reported that children from low-income families who displayed good academic outcomes were rare. Therefore, we used a snowballing method through the first few participants and tapped into the personal social networks of the researchers. All means were exhausted by the time a total of 10 children were recruited within the time frame for the study (eight boys and two girls). The 10 children in the study were from different ethnic groups: seven Chinese, two Malays, and one Indian. Institutional ethics approval (NUS-IRB-16–305) was granted and the consent by parents and assent by children were obtained before data collection (see Table I).

The researcher spent time to build trust and rapport with the children recruited for this study. Most of the children were shy and soft-spoken at first, but after a few rounds of games they appeared to be more comfortable and at ease. While introducing the study to the children, the researcher was careful not to take the position of an expert, but rather the stance of a curious researcher interested in the lives of the children and acknowledging children as experts of their own lives. Rapport with the children strengthened as the data collection progressed. The researcher met each child five times during the two-month period.

### Audio diary

To capture the day-to-day agentic expression of the PD children in real time, children were tasked to audio-record the challenges they faced in school, financial shortages, and dynamic relationships with their families at the end of every day. This method facilitated access to contextualized data. The audio-diary method has previously been used to study first-time mothers in their breastfeeding journey (Williamson, Leeming, Lyttle, & Johnson, 2012), daily experiences of young people with visual disabilities (Worth, 2009), and children’s resistance to parents’ rules (Kuczynski, Pitman & Twigger, in this issue). To the best of our knowledge, it has not been used with children from low-income families. The audio-diary method allows participants the choice of speaking for themselves and highlighting particular aspects of their daily experiences that are most meaningful to them (Bolger, Davis, & Rafaeli, 2003; Sandstrom & Cillessen, 2003).

Children were asked to maintain a digital audio diary for five consecutive days within a one-week period. In the diary guide, we asked children to report moments of happiness or sadness in the day and state the members in the family who had contributed to those moments. The purpose of these questions was to access the nature of interdependence and connectedness of children with the other family members within the *relationship contexts*. Children were also requested to describe one challenge that they faced in their school work and money issues in the day, and how they managed such challenges. From these descriptions, we hoped to gain insights into the *construction* and *action aspects of agency* of PD children when they encountered obstacles in meeting their goals.

### Semi-structured interviews

The audio-diary data served as a base for formulating context-specific interview questions that were targeted at each child. The researcher summarized the combined data from the audio diary and invited the children to elaborate on each of the 5 days with open-ended questions such as “Tell me what happened on Monday.” In addition, the researcher also brought up specific incidents from their diaries to seek clarification. For example, on the third day of her recording, Deviyah described a challenge she had encountered while doing her math homework. She recorded a statement: “I don’t understand, but I won’t stay in this position forever.” By following up on this statement, the researcher was able to gain insights into Deviyah’s determination and perspective in facing tough challenges. After clarifying salient points that stemmed from the audio diary, an in-depth interview was conducted by utilising an interview guide underpinned by SRT. Some of the open-ended questions invited children to discuss how they and their mothers envisioned their future. This question facilitated the examination of the construct of agency by giving children an opportunity to describe their perspectives of the parent-child *relationship context and also* the nature of their future goals for themselves. Finally, to reduce the sense of threat in discussing the topic of financial deprivation, *vignettes* were used for children to project onto a character their own experiences and thoughts on money constraints. We invited children to discuss how the lack of money might have implications on school performance when the character in the vignette could not afford private tuition or could not participate in social activities. These vignette questions were designed to assess children’s actions as agents in a comprehensive manner that included motivation, meaning making, and strategic action. The audio-diary method and the interviews worked synergistically to make space for the children’s free expression and allowed for a more in-depth exploration of their daily experiences (Williamson, Leeming, Lyttle, & Johnson, 2015).

### Data integration, interpretation, and analysis

The five-day audio diaries collected from the children were promptly transcribed and carefully combed through to pick up salient descriptors of agentic qualities expressed in their dealing with day-to-day challenges. These informed the research team regarding the aspects of agency to explore more deeply during the interviews. Once the interviews were transcribed, the analysis of transcripts from audio diary and interviews was performed through an iterative interpretation process for each participant. The researcher and the second author, who did not participate directly in the data collection, reflected critically and iteratively as a team on these two sources of data in order to deepen analysis. As many open codes (N = 75) as necessary were inductively created to capture the experiences of the children and their unique perspectives of their situations.

A higher level of integration of the two sources of data was conducted by importing all transcripts into the software Nvivo 11 which aided in inducting the coding of all data. The data were then triangulated and compared across participants to form axial codes (N = 8). Guided by SRT, the data were further categorized inductively into three selective codes to address the research questions: relationship contexts of PD children, motivation to perform well in school, and management of money problems. It became apparent as data analysis deepened that the selective code “relationship contexts of PD children” which captured the dynamics between PD and their mother provided a context for the other two selective codes. That is, the nature of mother-child relationship provides a context for the children to exercise agency—the motivation to do well and navigate through financial constraints. The eight axial codes were eventually integrated into two selective codes (See Table II).

**Table I.** Demographics of child participants.

Name of child (pseudonyms)	Gender	Age	Ethnicity	Type of financial assistance
Ashaf	M	11	Malay	^a^SPMF
Bo En	M	13	Chinese	SPMF/MOE FAS
Caleb	M	13	Chinese	^b^MOE FAS
Deviyah	F	10	Indian	SPMF
En Zhe	M	12	Chinese	SPMF/MOE FAS
Fadhil	M	10	Malay	SPMF
Gaby	F	10	Chinese	ComCare Student Care subsidies
Han Jie	M	12	Chinese	MOE FAS
Ian	M	12	Chinese	MOE FAS
Jun Ming	M	13	Chinese	MOE FAS

^a^SPMF: Straits’ Times School Pocket Money Fund is a pocket money assistance given to child/youth studying in Singapore. To receive the assistance, the per capita gross household income should not be more than S$625 an equivalent of USD$465.

^b^MOE FAS: Ministry of Education Financial Assistance Scheme is a scheme that serves to support children of families with per capita gross household income not exceeding USD$465.

**Table II.** Axial and selected codes for data analysis.

Relationship context	Axial code Code 1: nature of connectedness Code 2: child’s contribution to maintaining relationship Code 3: child’s interpretation of mother’s views
Motivation to do well	Axial code Code 1: child’s cognitive motivation Code 2: child’s actions and strategies to do well
Money issues	Axial code Code 1: child’s awareness of family financial status Code 2: child’s construction of meaning on money and spending Code 3: child’s good money sense

## Results

In this section, we report the two selective codes together with associated sub-codes: the PD children’s motivation for doing well in school, and their navigation through financial constraints. The two selective codes, together with associated sub-codes, can be found in Table II. The in-depth analysis revealed that the mother-child relationship context played an important part in motivating the children to do well in school. The relationship contexts seemed to facilitate the children’s expression of agency which resulted in strategies that bolster their motivation for doing well in school. The children’s agentic capabilities were not confined to school performance, but extended also to their navigation of the financial constraints in their family. They were able to redefine meanings of their financial circumstances and create new ways of maximizing their resources.

### Motivation to do well

Children reported that the main motivation spurring them to excel was their relationship with their mothers and consideration of their family’s financial situation. While discussing their visions for the future, children expressed their own dreams and aspirations for doing well in various careers, be it as a lawyer, a businessman, an actor, a teacher or other interesting roles.

Eleven-year-old Ashraf said: “In 10 years’ time, I want to study law to be [a] lawyer to help other people with cases and can earn more money for my family or mummy.” Bo En, on the other hand, wished to harness his interest in acting and become a professional actor up to earn more money for himself and his mother. Another participant, Han Jie (12), remarked: “When I am 22, I want to be doing business and get great success so that mum would feel worth it for all that she has done for us, and now I can finally give her money also.” A common thread running through all children’s narratives about their aspirations was that their motivation to do well was closely linked to their desire to give their families a better life.

#### Mother–child relationship as a source of motivation

The analysis of data on how children regarded happy and sad moments with their mothers showed that they shared close relationships with one another. The intimacy between children and mothers was not only reflected through common activities that they enjoyed together, but also was seen through the emotions expressed by the children as they talked about their mothers. When asked about how he viewed his mother, Han Jie (12) said his mother was a caring person who put his needs before her own.

“My mum is caring as she always considers our [children’s] feelings and doesn’t get angry with us even when we feel too tired to go out with her. She is also very joyful because I have never seen her cry; she always smiles even when things are tough.”

For Caleb (13), seeing his mother pick him up from the bus stop on a rainy day was the happiest moment for him as it reminded him of her care for him.

“It was a long day at school and I was all tired and sticky and it was raining, and I asked my mum to walk to the bus stop to help me with my books. I got happy just to see my mum waiting for me there patiently and to know how much she cares for me.”

Evidence of children’s connected relationships with their mothers, was not confined to children’s responses towards their mothers’ initiations and actions. Children also saw themselves as actively contributing to nurturing the relationship. For example, children reported that when their mothers initiated conversations about their day in school, they not only shared their experiences but also reciprocated by encouraging their mothers to talk about their experiences at work. Bo En (13) reported that his mother would disclose her struggles at work to him and he would encourage her and help her feel supported by waiting for her to return home from work.

“Sometimes, my mummy would share about her work and complain a bit, then I will just listen. Every night, I will try to wait for her to come back and we can have time to watch TV together so that she has someone to talk to, because we are like best friends.”

### Children’s interpretation of mother’s expectations on school performance

The close relationship shared between the children and their mothers also appeared to be a source of motivation for children. All the PD children in our sample said they worked harder in their studies because they felt supported by their mothers who encouraged them to “just do their best.” Children knew that their mothers had high expectations for their studies, and yet they did not feel undue pressure to perform. “My mum always says that she believes that I can get number one [in class] if I really work hard, but she doesn’t say I must definitely do well because she knows that I can eventually do it.” (Gaby, 10)

Children reported that their mothers’ encouragement and confidence in their abilities made them less stressed about school and motivated them to work harder in their studies.

“When my mother says just do your best and pass, I feel like I am happy and don’t feel stressed, but at the same time I also feel that I am better able to go and get my ‘A’s because I know I am capable of getting good results.” (Deviyah, 10)

#### Children’s own strategies to bolster motivation

Children reported that they actively thought about strategies to motivate themselves to work hard in their studies so as to achieve academically and improve their family’s lives. These strategies included achievement affirmations and extracurricular reading,

#### Achievement affirmations

One of the ways the children kept themselves going when they were tired or stressed from school was to use personal study quotes adapted from books or the Internet or improvised on their own. These affirmations were usually pinned to their walls or placed in their pencil cases. Some examples of the quotes used by the children include “there is always a success in life, if you want, just try, if you want, just do it!”, “work hard not smart”, and “you can do it!” Whether these affirmations whether borrowed from other sources or invented by the children themselves reflected the children’s beliefs and actions in motivating themselves consistently to press forward despite difficulties.

#### Reading

Although the children had limited financial resources and therefore did not own many books at home, five out of 10 of them regularly made use of the free resources from school or public libraries. The children saw reading as a means to de-stress, gain new perspectives and find inspiration to motivate themselves. For Ashraf (10), reading books helped him to stay calm and find the courage to face his fears. Children also reported that extracurricular reading had the additional benefit of increasing their vocabulary for use in their school assignments.

### Children’s navigations through financial constraints

Our analysis also shed light on how PD children navigated through their daily lives despite their limited financial resources. The following section describes the four subthemes of how the children navigated through the financial constraints in their home as shown in Table II. These subthemes include: their the awareness of their family’s financial status, their refusal to see their families as being poor, their keenness in helping with their family finances, as well as their ability to transform (redefine/reconstruct?) the meaning of receiving financial aid.

#### Awareness of family financial status

When asked about their family’s situation, all the children indicated that they were aware of their families’ financial struggles. Some children said that their parents had told them about the financial situation at home while others indicated that they inferred that their family was disadvantaged through observations from their daily lives. En Zhe (12) said that his mother tried not to let him worry about the financial situation by assuring him that they had a “lot of money”. However, En Zhe was an agent in making his own interpretations of his daily experiences. He was aware that the family was struggling with money and had little savings to tide them through times of emergency. Deviyah (10), learned about her family’s financial struggles through her mother’s open sharing and her own deduction from the knowledge of her parents’ jobs. “I have heard since young that my parents are working as a security guard and a cleaner and they don’t earn much. So I do understand that my family has fewer resources and may not have a lot of money.”

#### Refusal to see family as poor

Although the children were aware of their family’s impoverished financial situation, all said that they did not see their families as poor. Instead, most children described that their families were wealthy in other ways such as time for each other and love.

“Maybe to other people we may be poor, but my mum is working part time and can still provide for our family of 4… We also always make sure we have time to spend quality time together. So I don’t think that our family is low-income at all, because we are rich in quality time.” (Caleb, 13)

Despite being aware of their families’ financial struggles, children did not focus on lack of money. Instead, they created new meanings out of their experiences and circumstances to reframe their family situation in alternative, less tangible ways. Some of the children said they did not see their family as poor because all their basic needs were met and the family had sufficient resources. Some also made reference to less fortunate people because in comparison, they felt that they were more fortunate. “No, my family is not really low-income, and we are good because we can eat and have a roof over our heads. All of these that we have are enough for us. There are some people who don’t even have the money to buy food” (Han Jie, 12). The children’s narratives reflected their sense of contentment and gratitude and highlighted their focus on the things they had instead of what they were lacking. There was also a strong sense of pride as the children spoke about their families. Children highlighted that despite being labelled by society as “low-income” and being aware of their families’ financial struggles, they refused to see their families as poor or identify as low-income as it was not part of their lived experiences.

#### Management of desires

Although most children were content to have a roof over their heads and food on the table, four said that they did desire luxuries and money to buy their favourite toys. However, they also said that they did not insist, either because they had the family’s financial situation in mind or because they accepted their mother’s explanation as to why they were unable to get what they wanted. For example, Caleb said that his reflections on his mother’s advice helped him to accept the importance of delaying gratification.

“I really wanted that Lego set and I told my mum, and she said if you really want it, you have to use your savings to buy so I tried to save for two years. But eventually the set that I wanted was discontinued and then my mum told me that ‘hey you don’t really need it after all, cos you already saved two years without it’, and I thought carefully and realized that she was right … because I always desire for so many Lego sets, so I began to think that it was better to save more, since they are all about the same.” (Caleb, 13)

In this narrative, it can be seen that Caleb exercised his agency by reinterpreting his desire in light of his mother’s comments. It was not blind compliance but a willingness to accept explanations and rationales if it made sense to him. His awareness of the financial strain at home helped him to choose not to put his family at risk because of his own desires.

#### Helping with family finances

Knowing their family’s lack of financial resources, some children expressed their desire to help their families by saving more money. Children said they would save up their allowance and only use it for necessities such as food or school supplies, keeping the rest in their piggy banks so as to avoid requesting from their parents. Ashraf (10) shared that his mother used a “top-up” system and would give him an allowance of $2 per day whenever she checked that his wallet was empty. He would try his best to limit spending his money so that his mother would not need to replenish his allowance every day.

Deviyah (10) said she tried to save her allowance bit by bit every day. Her goals were both to avoid being a financial burden and also to help her mother with her own savings when she knew that the money situation was tight.

“Even if it is just a 50 cents [coin] I can save it, two 50 cents would make $1 and I can save so that I don’t have to ask money from my mother and can even pass her some money to use. Sometimes when there is not enough money for the bills, I would pass her some money and it makes me happy to be able to help.”

These narratives of connected agency by Ashraf and Deviyah are not isolated cases. The desire to save money on behalf of their family was reflected in seven out of the 10 children in this study. The children were motivated to save money for the family and were willing to give out of their own savings in order to help with bills and expenses. The joy that they derived from being able to help their mothers with bills was also striking, reflecting a sense of close connectedness between children and mothers.

Children discussed how they devised creative strategies to help them maximize the money they had. Gaby (10) shared that she did not eat at recess time on most days so that she could keep the meal coupons given to her for lunch.

“I don’t eat during recess because I already ate breakfast and I want to save money for lunch… I have a coupon every day at the Student Care… also one classmate at the back [of the class] always gives me food so I will just eat that because I don’t want to spend my money and waste money, so my mother does not have to give me more money and I can save more.”

Deviyah (10) also said that she made full use of free snacks given to her every day for breakfast as her meal for recess, so she could use her coupons for lunch rather than rely on her mother for pocket money.

En Zhe (12) maximized his resources by “helping” his friend purchase drinks through his EZ link card—[a cash card into which the financial aid for transactions in the school canteen is deposited] so that he could “cash out” the money when his friend paid him back in cash.

“I help my friend buy drinks [by using the EZ card] so I can change the money inside the EZ card [deposited by the school] into cash because now I don’t have any allowance [from my mother] at all, so since he [friend] is too lazy to buy I will frequently help him buy drinks so that he would pay me 50 cents in cash which I can save up and pass to mum to buy the groceries.”

The children’s accounts showed how they strategized and fully maximized their resources in order to stretch their money and use their savings to help their families.

#### Give new meaning to financial aid

Despite the social stigma attached to receiving financial assistance, the PD children neither chose to see it as something embarrassing nor felt the need to conceal it in front of their peers. Instead, most children mentioned that financial aid was beneficial for them because it helped them get food without tapping on their own savings. For them, financial assistance seemed like a resource that they could exploit.

“Eh free money [financial aid from school] in my EZ link card every day for lunch, why would it be something that I don’t dare to share with my friends? My friend even thought that I was rich because I got free money in my EZ link card. Some other friends even wanted to apply for it after seeing me use my EZ card. I would rather have it than not having it, [I] can keep my allowance [from my parents] as savings.”

This insight reflects the resourcefulness of the PD children not only in making the best use of financial assistance received but also in giving new meaning to the aid and altering their peers’ perception of what is commonly stigmatized.

## Discussion

For the Positive Deviant children in this study, growing up in poor families meant that they had to live with limited financial resources. The children, however, did not seem to consider such limitations as constricting their ability to do well. They were clearly not just recipients of their circumstances but active contributors to their families’ and their own well-being. From the data collected through the children’s audio diaries and interviews, and the careful analysis performed with the guidance of the sensitizing concepts of SRT, we recognized that the exceptional agentic capacities demonstrated by PD children must not be understood as mere individual traits or pursuits for individual goals. Instead, the children in our sample acted as connected agents whose actions were enabled by and carried out in a way that benefitted mutually important family relationships. These findings challenge the dominant discourses of disadvantaged children as victims and affirm the value of SRT in distilling agency in resource-deficit contexts (Chee, Goh, & Kuczynski, 2014).

### Relationship contexts that bolster PD children’s capacities as connected agents

Despite the shortage of money to attend extra tuition classes and other educational enrichment programmes, as well as the lack of parental coaching as their mothers had not received much education, these PD children performed well in school. Our findings cast doubt on the theory that such outstanding performance could be chiefly attributed to the individual child’s academic interests or traits (Constantine, Benard, & Diaz, 1999). Six out of the 10 children said that they were not particularly interested in school work, and yet worked hard and did well. We found supportive evidence that the relationship contexts, particularly between PD children and their mothers, might have a bolstering effect on the children’s sense of agency and motivation to work hard.

Close and mutually supportive relationships between PD children and their mothers were apparent in both sources of data reported by all the participants. Children reported that they knew their mothers had high academic aspirations for them and yet did not impose undue pressure on them. The evidence did not support a linear explanation of a “from-mother-to-children” influence such that mothers directly caused or transmitted to children the capacity for a good academic outcome. Instead the parent-child relationship context worked indirectly to support, constrain, and channel the children’s own efforts as agents. Children said the support they received from mothers’ trust that they would do their best and the encouragement they received helped them to achieve their own academic expectations and aspirations. Children did not feel compelled by their mothers to achieve but rather, the relationship contexts supported their own intentional efforts towards building a better future for themselves and their families.

We argue that these PD children were *connected agents* because they demonstrated the awareness and capacity to make a difference in the relationship with their mothers and families. The children’s thought processes were guided by consideration of their family’s financial constraints and not merely of achieving their personal goals. This was evident both in the way they motivated themselves to work hard as well as in the strategies they used to spend money within their means. The PD children believed that by achieving good academic results they were able to add something meaningful for their mothers, for themselves, and for the relationship (De Mol & Buysse, 2008; De Mol et al., 2018).

### Locally relevant solutions offered by PD children

The PD children in this study developed specific practices that, despite their adverse home conditions, enabled them to achieve better school outcomes. Their knowledge about the constant financial crunch which their families faced did not seem to elicit a sense of victimhood or resignation. A common thread among the PD children is that they did not regard themselves or their families as poor. Not only did they reject the label of “poor families” and therefore refuse the subtle image of “helplessness” that comes with being poor. Instead, they were able to articulate the strategies they developed to maximize and save money, delay gratifications and manage their material desires.

They even helped their mothers with family finances through their savings from the financial assistance they received from schools. In addition, they creatively reinterpreted the meaning of receiving financial assistance through schools, which might have been socially perceived by their peers as embarrassing or stigmatising, into something attractive which even provoked a sense of envy in their classmates from wealthier families.

The PD children’s agentic capacities and the knowledge (Goh & Baruch, 2018) they developed to resist the notions of “disadvantaged” and do well in school are valuable resources. Their success in attaining exceptional performance could be utilized to aid their counterparts in similar adversity to explore how and why things go right, and learn from their success (Baxter et al., 2015). Such solutions were internally generated by PD children and their families rather than externally imposed, hence ensuring feasibility and acceptability by their peers within similar contexts of financial limitation.

### Productivity of SRT in distilling evidence of agency

The rich data collected through audio diaries and interview with PD sample participants contained plentiful evidence of the role of children’s agency. The constructs proposed by SRT view children as strategic, interpretive agents helped identify specific actions and strategies that children undertook to achieve their goals and the meanings that children constructed to beneficially make sense of their environments. In addition, SRT’s proposal that agency must be understood in the context of long-term, interdependent relationship context, contributed to the identification of specific social supports that enabled children’s actions on behalf of themselves as well as their mothers and other family members.

### Implications of results for practice

This study yielded results that have valuable implications for practice by exploring the specific actions and contexts that make up child and family resilience.

The prudent use of limited money as well as strategies to maintain the motivation to work hard academically by PD children provided valuable examples of bottom-up solutions. In addition, the results on the nature of agency in relationship contexts gleaned in this study could expand the assessment and intervention strategies for social workers to improve the well-being of children from poor families. Awareness of the agentic capabilities of these PD children could shift the focus of social workers away from viewing children from low-income families as victims of their family circumstances, to capitalising on and supporting their strengths (Chee et al., 2014; Goh & Baruch, 2018). Also, instead of intervention solely at the individual level, either with children or mothers, the concept of relational agency proposed by SRT emphasizes the importance of interpersonal processes for constructing a person’s sense of agency (De Mol et al., 2018). Hence, intervention must be conceptualized at the levels of individual and relational contexts. Instead of a linear notion of treatment, with the assumption that the enhancement of family financial resources or the improvement in mothers’ parenting skills as “independent factors” would bring about improvement in the “outcomes” of the children, the results from this study recommend the relevance of tapping into or developing the relationship context between children and mothers or other significant adults in the family, so that the partners in the relationship experience a sense of interconnectedness. As children become connected agents, their confidence and capacity to exercise agency for themselves and their family could be enhanced. In addition, the successful behaviours and strategies gleaned from this PD sample may potentially inform behavioural change interventions that include skills transfer and monitoring the growth of children in similar family conditions (Lipping et al., 2002).

### Conclusion

We believe that the frameworks of positive deviance (PD) and social relational theory (SRT) complement each other in providing conceptual tool for exploring children’s contributions to their success in school, the well-being of their families, and children’s sense of agency in their lives. We acknowledge the small sample size of this study and the lack of generalizability of the findings. However, the strategy of identifying a small group of children who succeed academically despite adversity provided insight into the specific individual actions, thoughts and motives that constitute the agency of resilient children from low income families.
